# Nucleolar and Coiled-Body Phosphoprotein 1 Is Associated With Stemness and Represents a Potential Therapeutic Target in Triple-Negative Breast Cancer

**DOI:** 10.3389/fonc.2022.731528

**Published:** 2022-01-31

**Authors:** Sisi Chen, Ying Li, Muyao Wu, Lian Xue, Jianyu Zhu, Mi Wu, Qiuting Zhang, Guangchun He, Guifei Li, Shujun Fu, Chanjuan Zheng, Xiyun Deng

**Affiliations:** ^1^Key Laboratory of Model Animals and Stem Cell Biology in Hunan Province, Departments of Pathology and Pathophysiology, Hunan Normal University School of Medicine, Changsha, China; ^2^Key Laboratory of Translational Cancer Stem Cell Research, Hunan Normal University, Changsha, China; ^3^Department of Pathophysiology, Jishou University School of Medicine, Jishou, China; ^4^Department of Preventive Medicine, Hunan Normal University School of Medicine, Changsha, China

**Keywords:** triple-negative breast cancer, ribosome biogenesis, nucleolar and coiled-body phosphoprotein 1, stemness, targeted cancer therapy

## Abstract

Triple-negative breast cancer (TNBC) is the most aggressive subtype of breast cancer and lacks approved specific targeted therapies. One of the major reasons why TNBC is difficult to treat is the high proportion of cancer stem cells within the tumor tissue. Nucleolus is the location of ribosome biogenesis which is frequently overactivated in cancer cells and overactivation of ribosome biogenesis frequently drives the malignant transformation of cancer. Nucleolar and coiled-body phosphoprotein 1 (NOLC1) is a nucleolar protein responsible for nucleolus organization and rRNA synthesis and plays an important role in ribosome biogenesis. However, the correlation of NOLC1 expression with patient prognosis and its value as a therapeutic target have not been evaluated in TNBC. In the current study, based on bioinformatics analysis of the online databases, we found that the expression of NOLC1 was higher in breast cancer tissues than normal tissues, and NOLC1 was expressed at a higher level in TNBC than other subtypes of breast cancer. GSEA analysis revealed that stemness-related pathways were significantly enriched in breast cancer with high NOLC1 gene expression. Further analyses using gene expression profiling interactive analysis 2 (GEPIA2), tumor immune estimation resource (TIMER) and search tool for retrieval of interacting genes/proteins (STRING) demonstrated that NOLC1 was significantly associated with stemness in both all breast cancer and basal-like breast cancer/TNBC patients at both gene and protein levels. Knockdown of NOLC1 by siRNA decreased the protein level of the key stemness regulators MYC and ALDH and inhibited the sphere-forming capacity in TNBC cell line MDA-MB-231. Univariate and multivariate Cox regression analyses demonstrated that NOLC1 was an independent risk factor for overall survival in breast cancer. PrognoScan and Kaplan-Meier plotter analyses revealed that high expression of NOLC1 was associated with poor prognosis in both all breast cancer and TNBC patients. Further immunohistochemical analysis of breast cancer patient samples revealed that TNBC cells had a lower level of NOLC1 in the nucleus compared with non-TNBC cells. These findings suggest that NOLC1 is closely associated with the stemness properties of TNBC and represents a potential therapeutic target for TNBC.

## Introduction

Breast cancer is the most commonly diagnosed malignancy worldwide and the leading cause of cancer-related death in women (1). Triple-negative breast cancer (TNBC), which is characterized by the lack of expression of estrogen receptor (ER), progesterone receptor (PR), and expression/amplification of human epidermal growth factor receptor 2 (HER2), is the most aggressive and difficult-to-treat subtype of breast cancer (2). TNBC presents high probability of early recurrence and distant metastasis, leading to poor outcomes (3). To date, chemotherapy remains the mainstay of systemic therapy for TNBC, while the majority of patients after treatment develop recurrence within 5 years of diagnosis (4, 5). Recently, novel targeted therapeutic strategies for TNBC including immune checkpoint inhibition, PARP inhibition, and antibody-drug conjugates have shown benefit for some but not all TNBC patients (6). A subpopulation of cells with stem-like properties (stemness), called cancer stem cells (CSCs) (7), are considered responsible for tumorigenesis, therapy resistance, and relapse (8). Therefore, targeting CSCs is regarded as a promising therapeutic strategy for TNBC.

Nucleolus, a well-characterized membraneless subnuclear structure in the nucleus, has recently been investigated as a novel promising target for cancer therapy (9). Nucleolar and coiled-body phosphoprotein 1 (NOLC1, also known as NOPP140) is a 140 kDa nucleolar phosphoprotein that contains a nuclear localization signal-binding sequence (10). It is localized in the dense fibrillar component of the nucleolus and is a chaperone shutting between the nucleolus and the cytoplasm (10, 11). As a nucleolar protein, NOLC1 regulates rRNA transcription by interacting with the largest subunit of RNA Pol I (RPA194) (12). Overexpression of NOLC1 results in dysfunction of ribosome biogenesis, including the mislocalization of nucleolar proteins, improper formation of the nucleolus, and inhibition of rRNA transcription (12–14). Moreover, NOLC1 plays an important role in the regulation of tumorigenesis and synergistically co-regulates murine double minute 2 (MDM2) expression with TP53 in some types of cancer (15). In our previous study, we found that the protein level of NOLC1 in MDA-MB-231 breast CSCs was decreased in response to doxorubicin treatment (16), suggesting that NOLC1 might be a potential therapeutic target for TNBC. However, it’s not known whether NOLC1 expression is higher in TNBC than other breast cancer subtypes and whether it is correlated with the prognosis of TNBC patients.

**Figure 1:**
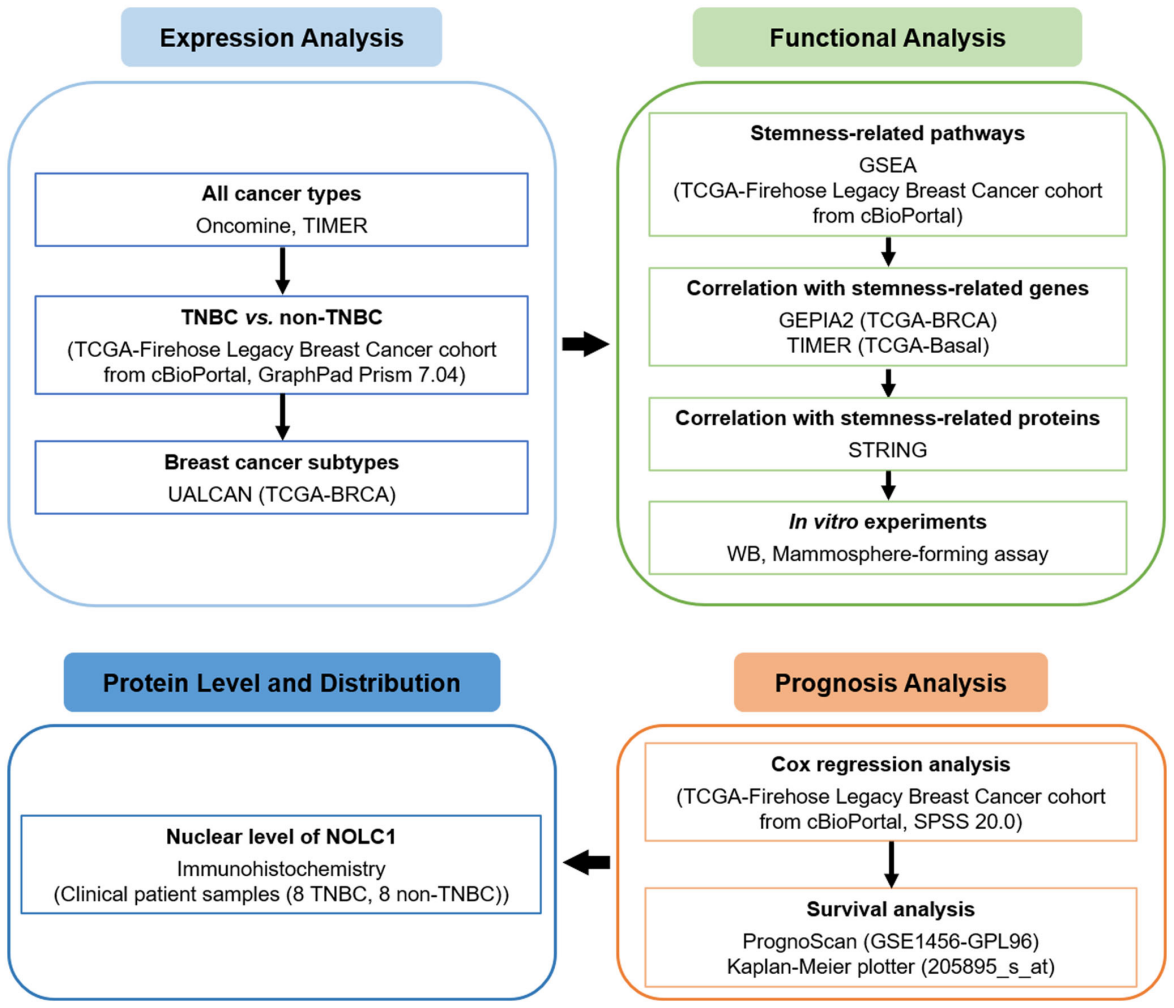
A flow chart showing the overall design of the study.

In the present study, we downloaded the TCGA dataset from the cBioPortal database and comprehensively analyzed NOLC1 expression in both all breast cancer and TNBC patients using web-based databases including Oncomine, tumor immune estimation resource 2.0 (TIMER2.0), and UALCAN. We also sought to evaluate the biological pathways involved in breast cancer with different expression levels of NOLC1 using gene set enrichment analysis (GSEA). Moreover, as we found stemness-related pathways were significantly enriched in breast cancer patients with high NOLC1 expression, we further investigated the correlation of NOLC1 with stemness-related genes *via* gene expression profiling interactive analysis 2 (GEPIA2), TIMER2.0, and search tool for retrieval of interacting genes/proteins (STRING). We explored the correlation between NOLC1 and stemness properties of TNBC by Western blot analysis and mammosphere-forming assay. Furthermore, we analyzed the relationship between NOLC1 expression and the prognosis of breast cancer patients by univariate and multivariate Cox regression, PrognoScan, and Kaplan-Meier plotter analyses. We also investigated the protein level and distribution of NOLC1 in TNBC and non-TNBC patients by immunohistochemistry. The flow chart of our study is illustrated in **Figure 1**. The findings in this study reveal a strong relationship between NOLC1 and stemness of TNBC and suggest that NOLC1 may be a potential therapeutic target in TNBC.

## Materials and Methods

### Data Collection

To explore the expression levels of NOLC1 in TNBC and non-TNBC, we analyzed the RNA-seq data of 115 TNBC and 982 non-TNBC clinical samples from the TCGA-Firehose Legacy Breast Cancer cohort downloaded from the cBioPortal database (http://www.cbioportal.org/study/summary?id=brca_tcga), which contains mRNA expression and clinical profiles. These data were also used for GSEA and Cox regression analysis below.

### Oncomine Database Analysis

We analyzed NOLC1 expression in different human cancer types by using the Oncomine database (https://www.oncomine.org/resource/login.html), which has become an industry−standard tool cited in > 1,100 peer−reviewed journal articles (17). The relevant statistical indicators we used were *P* < 0.05, fold change > 2, and gene ranking in the top 5%. The paired Student’s *t*−test was used to generate *P*−values to compare the expression differences between cancer and adjacent non−cancerous tissues.

### TIMER Database Analysis

Tumor immune estimation resource 2.0 (TIMER2.0) database (http://timer.cistrome.org/) (18–20) was used to further confirm the differential expression between cancer and adjacent non-cancerous tissues for NOLC1 across all TCGA tumors and the significantly correlated genes in GEPIA2. The Gene_DE module generated box plots to display the distributions of NOLC1 expression levels. The statistical significance computed by the Wilcoxon test was annotated by the number of stars (*P < 0.05, **P < 0.01, ***P < 0.001). The correlation module in TIMER2.0 generated the expression scatter plots between a pair of user-defined genes in a given cancer type, together with Spearman’s correlation coefficient and the estimated statistical significance. We chose the TCGA-BRCA and TCGA-basal datasets for analysis. NOLC1 was used for the x-axis as gene symbols, and related marker genes were represented on the y-axis as gene symbols. The gene expression level was displayed with log2 RNA-seq by expectation-maximization (RSEM). The strength of the correlation was determined using the following guide for the absolute value: “0.00–0.19, very weak”; “0.20–0.39, weak”; “0.40–0.59, moderate”; “0.60–0.79, strong”; “0.80–1.0, very strong”.

### UALCAN Database Analysis

To explore the NOLC1 expression in different subtypes of breast cancer, we used the UALCAN (http://ualcan.path.uab.edu) database (21). The Cancer Genome Atlas (TCGA) level 3 RNA-seq data related to 31 cancer types and clinical patient data were used in UALCAN. The *t*-test was performed using a PERL script with Comprehensive Perl Archive Network (CPAN) module “Statistics::TTest” (http://search.cpan.org/~yunfang/Statistics-TTest-1.1.0/TTest.pm) (21). We used the TCGA-BRCA dataset in UALCAN to analyze the mRNA expression of NOLC1 in subtypes of breast cancer.

### Gene Set Enrichment Analysis

Gene set enrichment analysis (GSEA) (https://www.gsea-msigdb.org/gsea/msigdb/index.jsp) is a computational method that determines whether an *a priori* defined set of genes show statistically significant differential expression between high and low expression groups (22). The differentially expressed genes were obtained by dividing breast cancer patients into high and low expression groups according to the median of NOLC1 gene expression. The phenotype labels were NOLC1^high^ and NOLC1^low^. Gene set permutations were conducted 1,000 times for each analysis. Gene sets with *P* < 0.05 and false discovery rate (FDR) < 0.25 were considered as enriched.

### GEPIA2 Database Analysis

The correlation of NOLC1 expression with the stemness-related markers in breast cancer was analyzed using the online database Gene Expression Profiling Interactive Analysis 2 (GEPIA2) (http://gepia2.cancer-pku.cn/#index) (23). GEPIA2 is an interactive web that includes 9,736 tumors and 8,587 normal samples from TCGA and the GTEx projects, which analyzed the RNA sequencing expression. Gene expression correlation analysis was performed for given sets of TCGA expression data. The Spearman method was used to determine the correlation coefficient.

### STRING Database Analysis

Search tool for retrieval of interacting genes/proteins (STRING) is a database of known and predicted protein-protein interactions (PPIs) (24). To reveal the interactions between NOLC1 and stemness-related proteins, ribosome biogenesis-related proteins including NOLC1 and stemness-related proteins were searched using STRING v.11.0 (https://string-db.org/) to construct the PPI network. In our database search, the species was set to “Homo sapiens”, the confidence score cutoff was set at 0.4, and other settings were set to default.

### Prognosis Analysis

Univariate and multivariate Cox regression was used to explore the role of NOLC1 expression in survival along with other clinical features [age, stage, tumor size (T classification), distant metastasis status (M classification), and lymph node status (N classification)]. High and low NOLC1 expression was determined based on the median values. The hazard ratio (HR) with 95% confidence interval (CI) and log-rank *P* were computed. The correlation between NOLC1 expression and survival in breast cancer patients was analyzed by the PrognoScan database (http://dna00.bio.kyutech.ac.jp/PrognoScan/) (25). The cohort we used was GSE1456-GPL96. Then, we analyzed the relationship between NOLC1 expression and prognosis of breast cancer patients by using the Kaplan-Meier plotter database (http://kmplot.com/analysis/) (26). The Affymetrix ID of NOLC1 we chose was 205895_s_at.

### RNA Interference and Cell Transfection

The expression of NOLC1 was silenced with siRNA oligonucleotides in TNBC cell line MDA-MB-231. The cells (5.0 × 10^4^) were seeded into each well of the 12-well plate (Corning, Jiangsu, China) in 1 mL growth medium (DMEM (Gibco, Shanghai, China) supplemented with 10% fetal bovine serum (Biological Industries, Israel)) and incubated at 37°C with 5% CO_2_. Scramble RNA was used as a non-targeting control (NC). The siRNA oligonucleotides (30 pmol/well) were transfected into cancer cells using Lipofectamine 3000 (Invitrogen, Carlsbad, CA) according to the protocol provided by the supplier. The expression level of NOLC1 was confirmed with Western blot analysis 48 h after transfection. NC and siRNA sequences were designed by the GenomeRNAi database (http://www.genomernai.org/) (27) and synthesized by Sangon Biotech Co., Ltd (Shanghai, China). The sequences are listed as follows: NC, sense: 5’-UUCUCCGAACGUGUCACGU-3’, antisense: 5’-ACGUGACACGUUCGGAGAA-3’; siNOLC1_1, sense: 5’-CCAAGAAUUCUUCAAAUAA-3’, antisense: 5’-UUAUUUGAAGAAUUCUUGG-3’; siNOLC1_2, sense: 5’-GGUCCCAGAGCGAAAGUUA-3’, antisense: 5’-UAACUUUCGCUCUGGGACC-3’; siNOLC1_3, sense: 5’-AAGAAGACUGUACCUAAA-3’, antisense: 5’-UUUAGGUACAGUCUUCUUG-3’.

### Western Blot Analysis

Cultured cells were lysed using 1× cell lysis buffer (Cell Signaling Technology, Danvers, MA, USA) with 1× protease inhibitor cocktail (Complete Mini, Roche, Mannheim, Germany) and 1 mM phenylmethanesulfonyl fluoride (Sigma-Aldrich) added. After centrifugation, the supernatants (whole cell lysates) were collected and quantified by the BCA (CWBIO, Beijing, China) protein quantification method. The lysates were mixed with the LDS sample buffer (Invitrogen, Carlsbad, CA) and reducing agent and denatured by boiling. The same quantity (5 μg) of protein from each sample was then separated on 10% denaturing PAGE gels followed by incubation with the respective primary antibody (NOLC1, Abcam, Cat#ab184550, 1:10,000; MYC, CST, Cat#5605, 1:1,000; ALDH, BD, Cat#611194, 1:1,000; β-actin, Bioworld, Cat#AP0060, 1:5,000) at 4°C overnight. The blots were incubated with the HRP-conjugated secondary antibody and the bands visualized by enhanced chemiluminescence according to our standard procedure (28).

### Mammosphere-Forming Assay

The cells were collected and suspended in breast cancer stem cell medium which consisted of DMEM/F12 (Life Technologies, Grand Island, NY, USA) supplemented with 1× B27 (Life Technologies), 20 ng/mL EGF (Prospec, East Brunswick, NJ, USA), 20 ng/mL bFGF (Prospec), 0.4% BSA (Sigma-Aldrich, St Louis, MO, USA), and 4 µg/mL Insulin [Genview, Pompano Beach, FL, USA)]. The cells were seeded at 5,000 cells/mL in breast cancer stem cell medium (100 μL) in the 96-well ultra-low attachment plate (Corning, Jiangsu, China). RNA interference was performed after cell seeding. The siRNA oligonucleotides (3 pmol/well) were transfected into cancer cells using Lipofectamine 3000 (Invitrogen, Carlsbad, CA) according to the protocol provided by the supplier. After 5 days, sphere-forming capacity was measured by calculating the areas of mammospheres formed as previously described (29).

### Immunohistochemistry Analysis

Sixteen paraffin-embedded tissue samples (8 TNBC and 8 non-TNBC, pathologically and immunohistochemically diagnosed) were obtained from female patients with breast cancer who had received surgeries in Xiangya Hospital, Central South University. Paraffin sections were baked in an oven at 60°C for 3 h, and then dewaxed in two changes of xylene for 10 min each, followed by hydration in graded ethanol for 3 min each. Endogenous peroxidase activity was quenched by 3% H_2_O_2_ for 20 min. For antigen retrieval, the slides were immersed into citric acid retrieval solution and heated in a pressure cooker, and cooled down at room temperature. After blocking of nonspecific binding, the sections were incubated with the NOLC1 antibody (Abcam, Cat#ab184550, 1:100) in a wet box at 4°C overnight. Next, the sections were incubated with biotinylated secondary antibody followed by incubation with the streptavidin-peroxidase conjugate (SP9000, ZSGB-BIO, Beijing, China) and color development using the DAB/H_2_O_2_ system (ZLI-9610, ZSGB-BIO, Beijing, China). The samples were rinsed with H_2_O before being dehydrated in a rising-concentration series of ethanol and placed in a xylene bath before mounting. The pictures were captured using 4× and 20× objectives of the Vectra Polaris imaging system (AKOYA, MA, USA). The staining data of nuclear signal intensity were obtained with inForm version 2.4.2 (AKOYA, MA, USA). After manual training of the software for recognition of the hematoxylin-stained nucleus and DAB-stained signal, the intensity scores were obtained from the software-designated staining intensity (0: no or marginal staining; 1: weak; 2: moderate; 3: strong). The cells with the lower (0 + 1) and higher (2 + 3) intensity scores were grouped and used to calculate the percentage of NOLC1^low^ and NOLC1^high^ cells, respectively.

### Statistical Analysis

All the quantitative data were presented as mean ± SEM. The Western blots were quantified using the ImageJ software. The role of NOLC1 expression in survival along with other clinicopathological parameters was analyzed by Cox regression using SPSS Statistics version 20.0 (IBM, Armonk, USA). The areas of mammospheres formed were analyzed using the Image Pro Plus software (Media Cybernetics, Rockville, MD, USA). Significance differences between the two groups were assessed using the two-tailed *t*-test. The difference of NOLC1 immunostaining intensity between TNBC and non-TNBC tissue samples was analyzed using the χ^2^ test. *P* < 0.05 was considered statistically significant.

## Results

### NOLC1 Expression Is Higher in TNBC Than Other Subtypes of Breast Cancer

**Figure 2:**
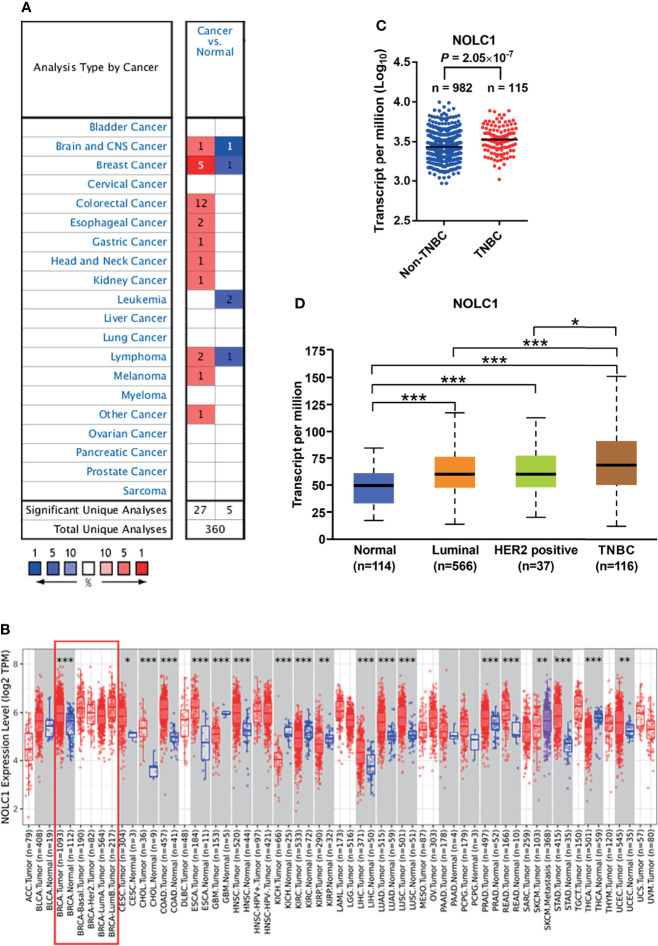
NOLC1 mRNA expression level in different types of human cancers and subtypes of breast cancer. **(A)** Higher or lower expression of NOLC1 in datasets of different cancers compared with normal tissues in the Oncomine database. The number in the graph indicates the count of datasets available for analysis, with red and blue representing high and low expression in tumor tissues compared with normal tissues, respectively. **(B)** NOLC1 expression levels in different human tumor types from TCGA determined by TIMER2.0 database analysis. The boxed area indicates comparisons between breast cancer and non-cancerous tissues and among different subtypes of breast cancer. **(C)** Expression of NOLC1 analyzed between TNBC and non-TNBC clinical samples from TCGA-Firehose Legacy Breast Cancer cohort. **(D)** NOLC1 mRNA expression levels in different subtypes of breast cancer analyzed by the UALCAN database. *P < 0.05, **P < 0.01, ***P < 0.001. TPM, transcripts per million.

To determine the differences of NOLC1 expression in tumor and normal tissues, the mRNA levels of NOLC1 in multiple cancer types were analyzed using the Oncomine database. We found that NOLC1 expression was higher in breast cancer tissues compared with normal tissues in 5 out of the 6 datasets available (**Figure 2A**). To further evaluate the mRNA expression level of NOLC1 in human cancers, we analyzed NOLC1 expression using the TIMER2.0 database. As shown in **Figure 2B**, NOLC1 expression was significantly higher in breast invasive carcinoma (BRCA) compared with adjacent normal tissues.

By analyzing the RNA-seq data of TNBC and non-TNBC samples from the TCGA dataset downloaded from the cBioPortal database, we found significantly higher levels of NOLC1 in TNBC compared with non-TNBC (**Figure 2C**). In order to further investigate the expression of NOLC1 in various subtypes of breast cancer, we used the UALCAN database. NOLC1 was found to be expressed at a higher level in TNBC than in any other subtype of breast cancer (**Figure 2D**). The median TPM value of NOLC1 in TNBC was about 1.15 times compared with either luminal (*P* < 0.001) or HER2-positive (*P* < 0.05) subtypes of breast cancer. These results suggest that NOLC1 expression might play an important role in TNBC tumorigenesis.

### Stemness-Related Pathways Are Enriched in Breast Cancer With High Expression of NOLC1

**Figure 3:**
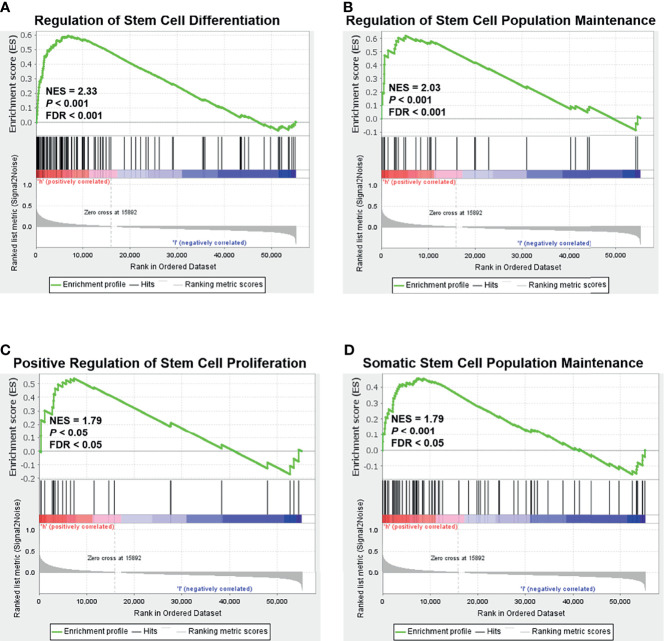
Gene set enrichment analysis (GSEA) according to the NOLC1 expression level in breast cancer. Significant enrichment plots of stemness-related pathways in breast cancer with NOLC1^high^ expression, including regulation of stem cell differentiation **(A)**, regulation of stem cell population maintenance **(B)**, positive regulation of stem cell proliferation **(C)**, and somatic stem cell population maintenance **(D)**. NES, normalized enrichment score.

As mentioned earlier, relapse and metastasis of TNBC are mostly due to CSCs (30). Based on the above findings, we speculate that NOLC1 might be related to the stemness properties of TNBC. To screen for stemness-related signaling pathways that were activated in breast cancer, we performed GSEA comparing the high and low NOLC1 expression groups. As shown in **Figure 3**, stemness-related pathways including regulation of stem cell differentiation, regulation of stem cell population maintenance, positive regulation of stem cell proliferation, and somatic stem cell population maintenance were significantly enriched in breast cancer with NOLC1^high^ expression. These results suggest that there is strong correlation between NOLC1 and stemness in breast cancer. Since the TNBC phenotypes are similar to stemness properties, we speculate that the high NOLC1 level might be associated with poor outcomes in breast cancer patients.

### NOLC1 Is Significantly Associated With Stemness in Basal-Like Breast Cancer

**Figure 4:**
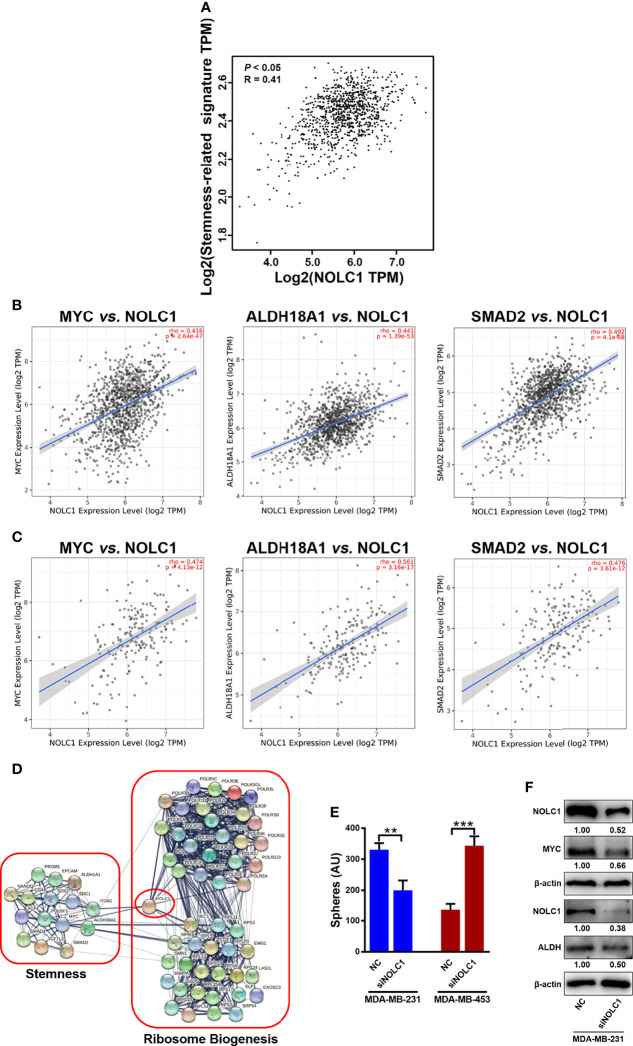
Correlation between NOLC1 and stemness in breast cancer patients and cells. **(A)** Correlation between NOLC1 and stemness-related gene set in breast cancer analyzed by GEPIA2 database. **(B, C)** Correlation between NOLC1 and stemness-related genes MYC, ALDH18A1, and SMAD2 in all breast cancer **(B)** and BLBC/TNBC **(C)**, respectively, analyzed by the TIMER2.0 database. **(D)** Protein-protein interaction network of ribosome biogenesis-related proteins including NOLC1 and stemness-related proteins in breast cancer. **(E)** Mammosphere-forming capacity of TNBC cell line MDA-MB-231 and non-TNBC cell line MDA-MB-453 after transfecting siNOLC1 detected by mammosphere-forming assay. **(F)** Protein level of MYC and ALDH after transfecting siNOLC1 into the TNBC cell line MDA-MB-231 detected by Western blot analysis. BLBC, basal-like breast cancer; MYC, MYC proto-oncogene; ALDH18A1, aldehyde dehydrogenase 18 family member A1; SMAD2, mothers against decapentaplegic homolog 2; TPM, transcripts per million. **P < 0.01; ***P < 0.001.

To further investigate the relationship between NOLC1 and stemness, we analyzed the correlation between NOLC1 and the stemness-related marker set in breast cancer using the GEPIA2 and TIMER2.0 databases. We found significant correlation between NOLC1 and the stemness-related gene set, including MYC proto-oncogene (MYC), aldehyde dehydrogenase 18 family member A1 (ALDH18A1), ALDH1A1, mothers against decapentaplegic homolog 2 (SMAD2), SMAD3, CD44 molecule (CD44), prominin 1 (PROM1, CD133), epithelial cell adhesion molecule (EpCAM), integrin subunit β1 (ITGB1, CD29), Nanog homeobox (NANOG), SRY-box transcription factor 2 (SOX2), transcription factor 7 like 2 (TCF7L2), POU class 5 homeobox 1 (POU5F1, OCT4), Kruppel like factor 4 (KLF4), and syndecan 1 (SDC1, CD138) in breast cancer through the GEPIA2 database (**Figure 4A**). Among these stemness-related genes, the top 3 genes positively correlated with NOLC1 were MYC, ALDH18A1, and SMAD2 in breast cancer when analyzed using the TIMER2.0 database (**Figure 4B**). We further explored the correlation between NOLC1 expression and the stemness-related genes in basal-like breast cancer (BLBC). Since BLBC is associated with the triple-negative phenotype, TNBC has been perceived as a synonym for BLBC (31). Correlation results showed the close association between NOLC1 and these stemness-related genes in BLBC (**Figure 4C**). In addition, the PPI network obtained using the STRING database revealed that the collection of ribosome biogenesis-related proteins including NOLC1 was correlated with stemness-related proteins through either direct or indirect interactions (**Figure 4D**).

In order to investigate the correlation between NOLC1 and stemness properties, we knocked down the expression of NOLC1 by transfecting siRNA into TNBC and non-TNBC cells. In agreement with our above-mentioned findings, knockdown of NOLC1 by siNOLC1 #3 inhibited the sphere-forming capacity of TNBC (MDA-MB-231) but not non-TNBC (MDA-MB-453) cells (**Figure 4E**). Furthermore, Western blot analysis revealed that knockdown of NOLC1 decreased the protein level of the key stemness regulators, MYC and ALDH, in MDA-MB-231 cells (**Figure 4F**). These findings suggest that NOLC1 may regulate the stemness-related pathway in BLBC or TNBC.

### NOLC1 Is an Independent Risk Factor and Is Negatively Correlated With Prognosis in Breast Cancer

**Figure 5:**
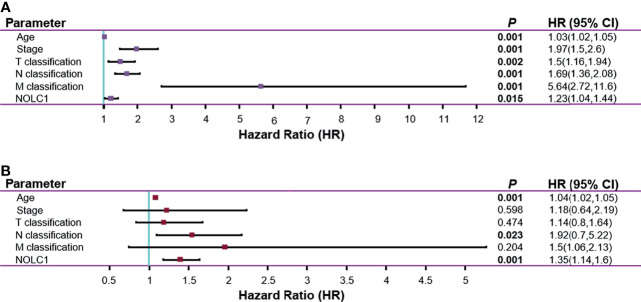
Univariate and multivariate Cox regression analysis for the expression of NOLC1 in breast cancer. Univariate **(A)** and multivariate **(B)** Cox regression analyses of clinicopathological parameters and NOLC1 in breast cancer. Bold values indicate *P* < 0.05.

Next, we investigated whether NOLC1 expression was correlated with prognosis in breast cancer patients. Univariate Cox analysis demonstrated that high NOLC1 expression was significantly associated with poor overall survival (OS) (HR = 1.23, 95% CI = 1.04–1.44, *P* = 0.015) (**Figure 5A****)**. Consistent with the results of univariate analysis, multivariate Cox analysis showed that high NOLC1 expression predicted poor OS of breast cancer patients (HR = 1.35, 95% CI = 1.14–1.6, *P* < 0.001) (**Figure 5B**). Therefore, it is conceivable that high NOLC1 expression is a potential independent risk factor for OS of breast cancer patients.

**Figure 6:**
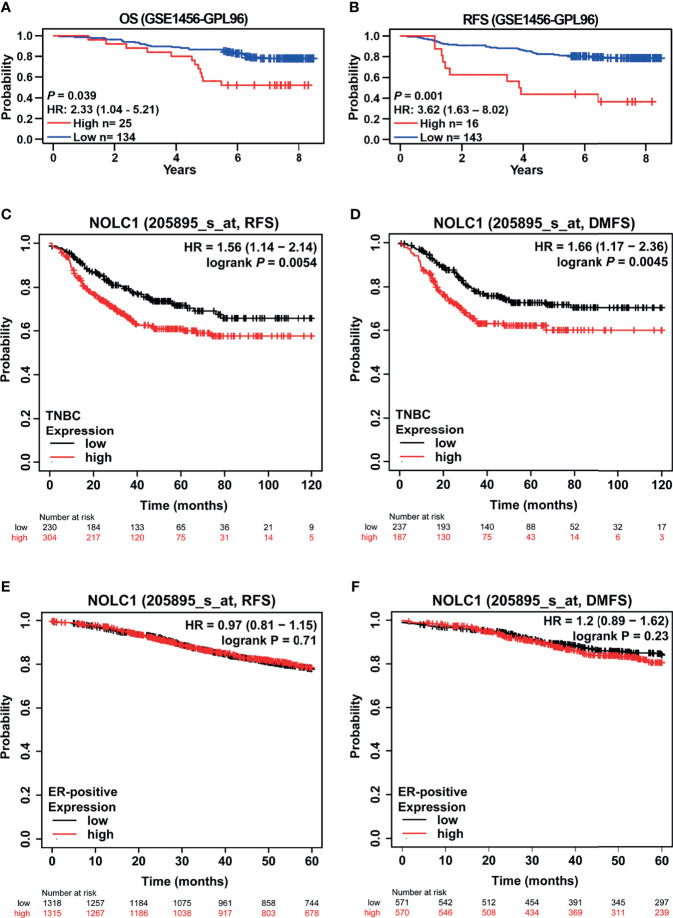
Kaplan-Meier survival curves comparing the high and low expression of NOLC1 in both all breast cancer and TNBC patients. Survival curves of OS **(A)** and RFS **(B)** in the breast cancer cohort (GSE1456-GPL97, n = 159) from the PrognoScan database. Survival curves of RFS **(C)** and DMFS **(D)** of TNBC from the Kaplan-Meier plotter database. Survival curves of RFS **(E)** and DMFS **(F)** of ER-positive breast cancer patients obtained from the Kaplan-Meier plotter database. OS, overall survival; RFS, relapse-free survival; DMFS, distant metastasis-free survival; HR, hazard ratio; CI, confidence interval.

The impact of NOLC1 expression on the survival of breast cancer patients was evaluated using the PrognoScan database. A cohort (GSE1456-GPL96) (32, 33) showed that high NOLC1 expression was associated with short OS and relapse-free survival (RFS) time (OS HR = 2.33, 95% CI = 1.04–5.21, *P* = 0.039; RFS HR = 3.62, 95% CI = 1.63–8.02, *P* = 0.001) (**Figures 6A, B****)**. We further analyzed the relationship between NOLC1 expression and prognosis of TNBC patients by using the Kaplan-Meier plotter database. We found that NOLC1 expression was negatively correlated with RFS and distant metastasis-free survival (DMFS) (RFS HR = 1.56, 95% CI = 1.14–2.14, log-rank *P* = 0.0054; DMFS HR = 1.66, 95% CI = 1.17–2.36, log-rank *P* = 0.0045) (**Figures 6C, D****)**. In addition, survival analysis of NOLC1 expression in ER-positive breast cancer patients showed no statistical difference (**Figures 6E, F****)**. We also performed survival analysis in HER2-positive breast cancer patients, which revealed that high expression of NOLC1 predicted better RFS in HER2-positive breast cancer (data not shown). These results suggest that high NOLC1 expression predicts poor prognosis in both all breast cancer and TNBC patients.

### Nuclear NOLC1 Level Is Lower in TNBC Than Non-TNBC Patient Tissues

**Figure 7:**
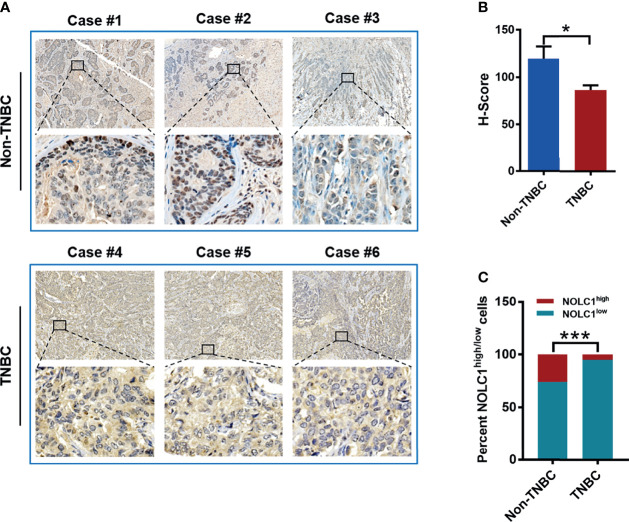
Protein level and cytoplasmic/nuclear distribution of NOLC1 in TNBC and non-TNBC patient tissues. **(A)** Representative immunohistochemical images showing the staining of NOLC1 in TNBC and non-TNBC tissue samples. The larger image from a portion of the tissue was illustrated at the bottom of each original image. Original magnification: 20×. **(B)** Summary of the H-Score of NOLC1 in the nucleus of TNBC and non-TNBC samples. **(C)** Distribution of the NOLC1^high/low^ expression in the nucleus of TNBC and non-TNBC tissue samples. *P < 0.05; ***P < 0.001.

In order to establish the clinical relevance of NOLC1 for TNBC patients, we performed immunohistochemical staining of NOLC1 in tissue samples derived from 8 TNBC and 8 non-TNBC cases. **Figure 7A** shows representative immunohistochemical staining of NOCL1 in TNBC and non-TNBC patient tissues. Quantification of the staining intensity revealed that the protein level of nuclear NOLC1 was significantly lower in tissues of TNBC patients compared with non-TNBC patients **(****Figure 7B****)**. Furthermore, the percentage of NOLC1^high^ cells was significantly lower in TNBC samples than that in non-TNBC samples **(****Figure 7C****)**. These results, although different from the above online-based bioinformatics results, suggest that NOLC1 in the nucleus is indeed differentially expressed between TNBC and non-TNBC patients.

## Discussion

TNBC is a highly aggressive and refractory subtype of breast cancer. The treatment of TNBC remains a clinical challenge due to the lack of effective therapeutic targets, leading to limited response and eventual resistance to existing treatment regimens (34). Several studies have reported that the resistance of TNBC to chemotherapy is mainly due to the fact that most of the tumor cells derived from BLBC or TNBC are CSCs (35, 36). Therefore, exploring specific therapeutic targets for TNBC CSCs is an urgent task. Increasing evidence has suggested the nucleolus is a novel therapeutic target for cancer (9). The canonical function of the nucleolus is ribosomes biogenesis, and several findings have shown that the pathways involved in ribosome biogenesis are frequently overactivated in cancer (37, 38). This phenomenon has led to an important conceptual advancement in cancer therapeutic strategy, especially for the development of targeted therapies (39). In our study, GSEA showed that the stemness-related pathways were significantly enriched in breast cancer with high NOLC1 expression. Further analyses using the GEPIA2 and TIMER2.0 databases revealed that NOLC1 was positively correlated with stemness-related genes, including MYC, ALDH18A1, ALDH1A1, SMAD2, SMAD3, etc., in both all breast cancer and TNBC. Moreover, results obtained from protein-protein interaction analysis also showed that ribosome biogenesis-related proteins including NOLC1 interacted with markers of stemness in breast cancer. Moreover, knockdown of NOLC1 inhibited the mammosphere-forming capacity and decreased the protein level of MYC and ALDH in TNBC cells. These results indicated that NOLC1 was a major link between ribosome biogenesis and stemness in TNBC. Therefore, simultaneous targeting ribosome biogenesis and stemness may be a novel therapeutic strategy for TNBC.

NOLC1 plays an important role in nucleologenesis by maintaining the fundamental structure of the fibrillar center and dense fibrillar component in the nucleolus (40). It also affects ribosomal processing and modification by regulating RNA Pol I (12, 41). A previous study has shown that NOLC1 is expressed at a higher level in nasopharyngeal carcinoma tissues than in normal tissues and is involved in tumorigenesis (15). In contrast, NOLC1 expression is lower in human hepatocellular carcinoma tissues compared with the matched non-cancerous tissues, and overexpression of NOLC1 suppresses cell proliferation of hepatocellular carcinoma (42). According to several studies, NOLC1 localized in the nucleolus interacts with telomeric repeat-binding factor 2 (TRF2), and the overexpression of NOLC1 inhibits cell proliferation by inducing apoptosis and cell cycle arrest in hepatocellular carcinoma (14, 43). In addition, NOLC1 also plays a role in the development of prostate cancer and is highly expressed in cancer tissues (44). In our study, bioinformatics analysis of high throughput RNA-sequencing data from TCGA revealed significantly increased NOLC1 expression in breast cancer tissues compared with the adjacent breast tissues. As expected, NOLC1 was expressed at a higher level in TNBC than other subtypes of breast cancer. Additional bioinformatics and experimental analyses revealed a close relationship between NOLC1 and the stemness-related pathway in TNBC. These findings point to a previously unidentified role for NOLC1 in TNBC tumorigenesis, which is worthy of further in-depth investigation.

Univariate and multivariate Cox regression analysis of TCGA dataset indicated that the high NOLC1 expression might be a potential independent biomarker for OS in breast cancer. Kaplan-Meier survival curves revealed that high expression of NOLC1 was associated with poor prognosis in both all breast cancer and TNBC patients. However, in non-TNBC patients, high NOLC1 expression was not correlated with poor prognosis. To further reveal the clinical relevance of NOLC1 expression, we performed immunohistochemical analysis of NOLC1 on paraffin sections derived from TNBC and non-TNBC patients. Contrary to our expectation, the protein level of nuclear NOLC1 was lower in TNBC compared with non-TNBC patient tissues. Even considering the cell as a whole (nucleus plus cytoplasm) (**Figure 7**), the level of NOLC1 was significantly lower in TNBC *vs.* non-TNBC (data not shown). The seemingly contradictory results between immunohistochemical staining and online bioinformatics analyses may simply reflect the difference between the protein and mRNA expression levels. While this possibility needs to be further investigated, the lower level of nuclear NOLC1 protein in TNBC *vs.* non-TNBC endorses our recent finding that lovastatin, a lipophilic statin identified by us as being able to inhibit EMT and metastasis of TNBC (45), could increase the nucleolar level of NOLC1 in TNBC cells (16). It is thus postulated that enhancing the nuclear/nucleolar level of the NOLC1 protein may result in the reversal of the TNBC phenotype towards that of non-TNBC. Nevertheless, this postulation needs to be validated using more sophisticated experimental models and, preferably, through investigator-initiated clinical studies. Once established, enhancing nuclear/nucleolar NOLC1 might have therapeutic potential with the benefit of improving TNBC patient survival. In summary, through comprehensive analyses of multiple online databases and investigations on cells and patient tissue samples, we provide strong evidence that NOLC1 is closely associated with the stemness properties of TNBC and represents a potential therapeutic target for TNBC.

## Data Availability Statement

Publicly available datasets were analyzed in this study. This data can be found here: http://www.cbioportal.org/study/summary?id=brca_tcga.

## Author Contributions

SC and CZ participated in the collection, analysis, and manuscript drafting. YL, MYW, LX, JZ, MW, QZ, GH, GL, SF, and XD reviewed the manuscript. CZ and XD conceived and designed the research. All authors have read and approved the final submitted manuscript.

## Funding

This work was supported by the National Natural Science Foundation of China (82173374, 81872167, 81472496); Key Grant of Research and Development in Hunan Province (2020DK2002); and Natural Science Foundation of Hunan (2019JJ40193, 2020JJ5386).

## Conflict of Interest

The authors declare that the research was conducted in the absence of any commercial or financial relationships that could be construed as a potential conflict of interest.

## Publisher’s Note

All claims expressed in this article are solely those of the authors and do not necessarily represent those of their affiliated organizations, or those of the publisher, the editors and the reviewers. Any product that may be evaluated in this article, or claim that may be made by its manufacturer, is not guaranteed or endorsed by the publisher.
